# Assessment of Bridging Stents in In Situ Laser Fenestrations of Aortic Endografts With Intravascular Ultrasound

**DOI:** 10.1016/j.ejvsvf.2024.05.008

**Published:** 2024-05-23

**Authors:** David Lindström, Anders Wanhainen, Kevin Mani, Giuseppe Asciutto

**Affiliations:** a Department of Surgical Sciences, Section of Vascular Surgery, Uppsala University, Uppsala, Sweden; b Department of Clinical Science and Education, Karolinska Institutet, Södersjukhuset, Stockholm, Sweden; c Department of Surgical and Peri-operative Sciences, Surgery, Umeå University, Umeå, Sweden

**Keywords:** Aortic endografts, Bridging stent, In situ laser, Intravascular ultrasound, IVUS

## Abstract

**Objective:**

Treatment of complex aortic aneurysms with the *in situ* laser fenestration (ISLF) technique involves implantation of a balloon expandable stent graft (bSG) in the created fenestration. Adequate expansion of this bSG is of importance both to achieve seal and to ensure target vessel stability. This experimental study assessed the expansion rate of different bSGs in the ISLF setting using intravascular ultrasound (IVUS).

**Methods:**

A commercially available aortic endograft was used to test the laser fenestration technique (Zenith Alpha, Cook Medical LLC, Bloomington, IN, USA). The ISLF was stented with the following bSGs: two Gore Viabahn VBX balloon expandable endoprostheses (WL Gore & Associates, Bloomington, IL, USA), three BeGraft Peripheral and three BeGraft Plus (Bentley InnoMed GmbH; Hechingen, Germany), and three Advanta V12 (Atrium, Hudson, NH, USA). The bSGs were expanded in three steps: (1) nominal, (2) rated burst pressure, and (3) dilation with a non-compliant balloon at 15 atmospheres. After each step, an IVUS assessment of the bSG minimum diameter and the area at the fenestration (FA) and in a fully expanded segment distal to the fenestration (SA) was performed. A mean of the three IVUS measurements was used as the value for comparison. An insufficient bSG expansion was defined as a mean of FA/SA of <0.8 (i.e., <80% expansion).

**Results:**

The VBX was the only bSG that could be expanded to its intended diameter (i.e., at least 80%) at nominal pressure. The BeGraft Peripheral and BeGraft Plus had the lowest degree of expansion after nominal and rated burst pressure. All bSGs that were tested reached a sufficient expansion degree after using a higher pressure balloon.

**Conclusion:**

In this *ex vivo* experiment, dilation up to nominal pressure showed satisfactory expansion only for the VBX. The consistency of the results when applied to the different types of stent grafts that were analysed reflects structural stent graft specific issues to consider when choosing the right device in cases of ISLF.

## Introduction

*In situ* laser fenestration (ISLF) of aortic endografts is a novel endovascular technique that is being used increasingly in the emergency setting.^1^, ^2^, ^3^ The technique of ISLF consists of three separate steps: creation of a fenestration by perforating the fabric at the level of the target vessel; pre-dilation using non-compliant balloons; and stenting of the target vessel using bridging stent grafts (bSGs).^4^^,^^5^ Balloon expandable stent grafts are commonly used in the setting of ISLF, to ensure an adequate seal in the fenestration and allow for dilation of the bridging stent to the diameter of the target vessel.

Even if this technique has mainly become standardised, there are still concerns about some of the technical options when performing an ISLF. Results of experimental data on stent grafts and balloon pressures to achieve an optimal seal without residual stenosis have been published previously.^6^ During these experiments, frequent and significant remaining stenoses in the bSG were noted when deploying the bSG using the pre-mounted balloon and nominal pressure during deployment. However, the residual stenosis degree could not be quantified. One outstanding question is whether the use of an additional higher pressure balloon can be avoided (to decrease ischaemia time) or if the pre-mounted balloon can result in sufficient expansion of the bSG.

This experimental study used intravascular ultrasound (IVUS) to assess the expansion rate of the bSG through the fenestration after inflation at nominal and rated burst pressure, and after using an additional higher pressure balloon.

## Methods

### Technique

One commercially available thoracic aortic stent graft proven to work in the ISLF setting^2^^,^^6^ was used to test the laser fenestration technique (Zenith Alpha, Cook Medical LLC, Bloomington, IN, USA). The aortic stent graft was submerged in saline at 37 ºC to create fenestrations using a 308 nm CVX-300 Excimer laser system (Spectranetics, Colorado Springs, CO, USA). The laser system was fitted with a 2.3 mm diameter Turbo-Elite laser catheter (Philips, Amsterdam, The Netherlands), which is compatible with an 0.018” guidewire. The settings used during this experiment were fluence 30 mJ/mm^2^ and rate 25 pulses/second. The bSG tested were: two Gore Viabahn VBX balloon expandable endoprostheses, 9 mm (WL Gore & Associates, Bloomington, IL, USA), three BeGraft Peripheral and three BeGraft Plus, 8 mm (Bentley InnoMed GmbH; Hechingen, Germany), and three Advanta V12, 9 mm (Atrium, Hudson, NH, USA). The choice of 8 mm and 9 mm stent grafts was primarily based on clinical practice and the average diameter of the target visceral vessels that are used with cases of ISLF, with the exception of the renal arteries.

**Figure 1:**
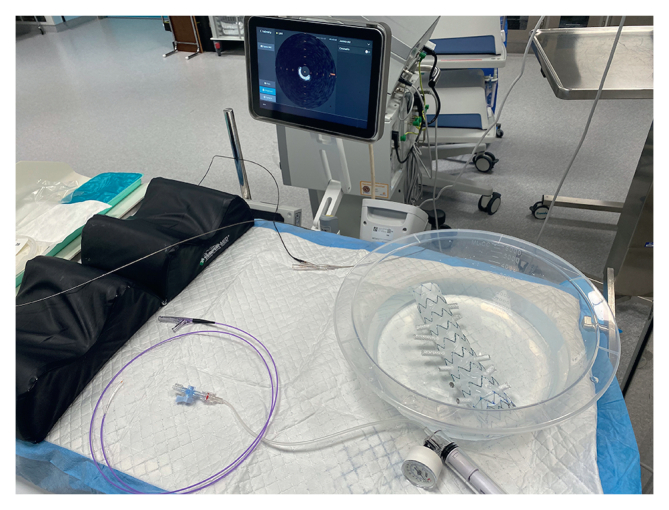
Photograph of the experimental setting.

Briefly, the laser fenestration was pre-dilatated with an Armada 6 x 40 mm plain balloon (Abbott Vascular, Santa Clara, CA, USA) at nominal pressure. After that, the bSGs were deployed across the fenestration and inflated to nominal and rated burst pressure according to the respective stent manufacturers. An Armada 8 x 40 mm (or 9 x 40 mm, depending on stent diameter) plain balloon was then inflated at 15 atmospheres (Fig. 1). The bSGs and ISLFs in the aortic graft were assessed visually at each step for the presence of any fabric tear (no further testing for tears was performed).

Furthermore, an IVUS assessment of the stent graft minimum diameter, the area at the fenestration, and in a fully expanded segment distal to the fenestration was performed. The IVUS assessment was performed three times for every bSG. A mean of the three IVUS measurements was used as the value for the comparison between the diameters and the area at the fenestration and the area distal to the fenestration (fenestration area, FA/stent graft area, SA). Insufficient bSG expansion was defined as a mean of FA/SA of <0.8 (i.e., <80% expansion).^7^

## Results

**Figure 2:**
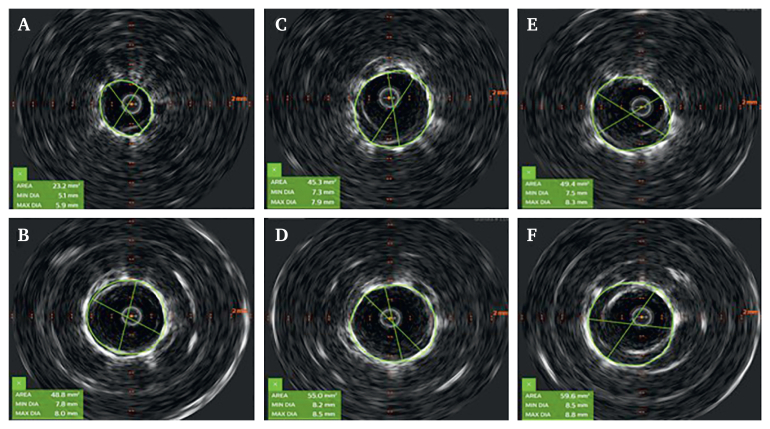
IVUS images. BeGraft Peripheral #1. At the height of fenestration and at a reference segment after nominal pressure (A, B), rated burst pressure (C, D) and high pressure (E, F).

The results are summarised in Table 1, Table 2. Of all 11 bSGs that were tested, only both VBX could be expanded to its intended diameter without the use of a higher pressure balloon. Two of three Advanta reached a sufficient expansion degree (i.e., at least 80%) at nominal pressure based on the area measurements. In the case of the Advanta #1, the regression to an insufficient expansion after ballooning at rated burst pressure was the result of an over expansion distal to the fenestration. The BeGraft Peripheral and the BeGraft Plus showed the lowest expansion degrees after nominal and rated burst pressure (Fig. 2). All tested bSGs reached a sufficient expansion degree after using a higher pressure balloon (15 atm). None of the bSGs and none of the fenestrations in the thoracic endovascular aortic repair graft showed any signs of fabric tears.

**Table 1:** Minimum diameter of the bridging stent grafts at fenestration.

Stent graft	Nominal pressure		Rated burst pressure		High pressure	
	Minimal diameter	% of intended	Minimal diameter	% of intended	Minimal diameter	% of intended
Advanta #1 (9 mm)	7.3	81	7.4	82	8.5	94
Advanta #2 (9 mm)	7.5	83	8.0	89	8.5	94
Advanta #3 (9 mm)	6.9	77	7.9	88	8.6	96
BeGraft Peripheral #1 (8 mm)	5.0	62	7.3	91	7.8	97
BeGraft Peripheral #2 (8 mm)	4.7	59	7.2	90	7.6	95
BeGraft Peripheral #3 (8 mm)	5.2	65	7.3	91	7.8	97
BeGraft Plus #1 (8 mm)	5.2	65	6.6	82	7.4	92
BeGraft Plus #2 (8 mm)	4.8	60	6.6	82	7.6	95
BeGraft Plus #3 (8 mm)	4.9	61	6.8	85	7.5	94
VBX #1 (9 mm)	8.4	93	8.8	90	9.3	103
VBX #2 (9 mm)	8.2	91	9.0	100	9.3	103

In brackets the intended diameter of the bridging stent graft. Reported values are the mean (mm) of three measurements.

**Table 2:** Stent graft area at the fenestration in relation to expanded segment distal to the fenestration.

Stent graft	Nominal pressure		Rated burst pressure		High pressure	
	Area fen/ref	Expansion	Area fen/ref	Expansion	Area fen/ref	Expansion
Advanta #1	44.5/54.0	82	45.8/61.1	75	61.3/63.8	96
Advanta #2	46.7/56.8	82	53.6/61.6	87	64.7/66.0	98
Advanta #3	39.7/55.7	71	53.0/61.6	86	62.0/65.9	94
BeGraft Peripheral #1	22.3/50.5	44	44.7/58.1	77	50.4/61.5	82
BeGraft Peripheral #2	20.1/44.7	45	44.7/57.3	78	51.0/62.2	82
BeGraft Peripheral #3	23.5/48.9	48	43.6/55.9	78	51.2/60.2	85
BeGraft Plus #1	23.2/43.8	53	36.6/50.1	73	46.0/54.1	85
BeGraft Plus #2	19.5/44.3	44	36.5/51.4	71	48.7/54.1	90
BeGraft Plus #3	20.3/41.4	49	38.4/49.2	78	48.4/56.3	86
VBX #1	59.4/66.0	90	64.2/72.9	88	79.1/79.9	99
VBX #2	55.6/67.8	82	67.8/77.9	87	72.9/74.4	98

Area values are the mean of three measurements of minimum area (mm^2^) of the bridging stent graft at fenestration. Expansion (%): area at fenestration/area distal to the fenestration. See text for explanation.

## Discussion

In this *ex vivo* experiment, dilation up to nominal pressure showed satisfactory expansion (i.e., at least 80% of the area of the fully expanded region distal to the fenestration) only for the VBX balloon expandable stent graft (bSG). For Advanta, BeGraft Peripheral, and BeGraft Plus, a further dilation up to 15 atmospheres was needed to achieve sufficient fenestration expansion. Even after the rated burst pressure dilation with the pre-mounted balloon there is a risk of leaving residual stenosis in the fenestration.

Pre-dilation of fenestrations should not be performed with >8 mm balloons, according to experimental data, since larger balloons increase the risk of fabric tears.^8^ The avoidance of a second balloon in the bSG is an obvious advantage, since it reduces further vessel manipulation and visceral ischaemia time. Furthermore, reaching sufficient expansion of the bSG is paramount since residual stenosis can result in instability and possible occlusion. The results of this experimental study suggest the use of a high pressure balloon when not using the VBX as the bSG for ISLF.

*In situ* laser fenestration is an option that can be considered in the emergency setting when a custom made device is not available. Excellent technical success can be achieved with this technique in experienced aortic centres.^9^ However, there are still concerns regarding the long term stability of bSGs used through ISLF.^1^ Ideally, a custom made device with reinforced fenestrations would also be used in emergency cases, but manufacturing times for these devices are still an issue. When looking into different acute options, some experimental testing^8^^,^^10^ and bench testing in this centre^6^ suggest that ISLF could be a valid option in emergency settings.

From a technical perspective, the selection of a polyester aortic stent graft was based on experimental^6^ and clinical studies.^1^ Regarding the laser equipment, a 2.3 mm filament was chosen to allow the passage of an 0.018” wire; this enabled a single six mm pre-dilation and subsequent bSG deployment. Another important aspect to consider when choosing a bSG to place through an ISLF is that of the different structure of the available bSGs on the market. The current study tested four different bSGs. The Advanta, the BeGraft Peripheral, and the BeGraft Plus have a continuous stent structure that is different from the VBX. This means that, at least theoretically, the VBX has a higher risk of instability when it happens to land with an unsupported segment at the height of the ISLF. This study did not look at this specifically. However, to date, one case of fracture of a VBX placed through an ISLF for the left subclavian artery has been observed (case report submitted).

This study also used a standardised method to perform the ISLF, choosing percutaneous transluminal angioplasty balloons compatible with the used bSG in terms of diameter, as well as a standard (15 mmHg) inflation pressure for the post-dilation. Furthermore, the outcome that was focused on was the bSGs expansion rates based on their intended diameter. These measures possibly limited but not completely excluded the fact that different stent diameters and structures could have influenced the results.

The experimental *ex vivo* design of the current study and the use of a single aortic endograft (other brands may require different pressures to remove any residual stenosis) should be seen as limitations of this study. Furthermore, the limited number of stent grafts that were examined resulted in the non-generalisability of the findings. However, the consistency of these results when applied to the different bSG types that were analysed reflect structural bSG specific issues to consider when choosing the right device in cases of ISLF.

There is a significant risk of residual stenosis in the bSG in ISLF if the dilation of the stent graft is performed only with the pre-mounted balloon. In this study, only the VBX stent graft achieved full expansion, whilst full expansion could only be secured when using a non-compliant balloon at 15 atmospheres in other brands. Careful evaluation of the bSG expansion should be performed during ISLF.

## Conflict of interest

None.
